# The Effect of Changes in the Separation Process for the Performance of Recycled Cement Powder: A Comparison with a Previous Study for Radioactive Waste Immobilization

**DOI:** 10.3390/ma15227972

**Published:** 2022-11-11

**Authors:** Ji-Hyun Kim, Eun-A Seo, Do-Gyeum Kim, Chul-Woo Chung

**Affiliations:** 1 Multidisciplinary Infra-Technology Research Laboratory, Pukyong National University, Yongso-ro 45, Nam-gu, Busan 48513, Korea; 2 Korea Institute of Civil Engineering and Building Technology, Goyang-daero 283, Ilsanseo-gu, Goyang-si 10223, Korea; 3 Division of Architectural and Fire Protection Engineering, Pukyong National University, Yongso-ro 45, Nam-gu, Busan 48513, Korea

**Keywords:** nuclear power plant, concrete, separation, recycled cement powder, radioactive waste

## Abstract

Separation of hydrated cement paste from aggregate is a key technology to reduce the amount of radioactive concrete waste during the decommissioning process. If separated cement-paste portions can be recycled as a solidifying agent for other radioactive waste, the amount of radioactive concrete waste could be close to “zero”. A study was conducted to achieve circular economy in the area of concrete decommissioning and found it to be successfully used as a solidifying agent for immobilization of liquid radioactive waste. However, previous work used a process that requires large amounts of energy (heat treatment was applied to most of the concrete fraction) because the objective was to completely remove hydrated cement powder from the aggregate. In this work, the separation system was modified to increase energy efficiency (heat treatment was applied to separated powder only), but such a change decreased the surface area of the recycled cement powder due to a higher inclusion of aggregate powder. A relatively lower solution to binder ratio could have been achieved for the preparation of wasteform specimens, and as a result, a 28 day compressive strength of wasteform could have become higher, but the final leachability indices were lower than the results observed from previous work. The results from 28 day compressive strength, thermal cycling and 90 day leaching experiments met the acceptance criteria for wasteform, indicating that this modified system can also be used for immobilization of liquid radioactive waste to meet the “zero” production of concrete waste during the decommissioning of a nuclear power plant. It should be noted that accurate monitoring of aggregate content in recycled cement powder during production is important to maintain proper reactivity of recycled cement powder.

## 1. Introduction 

The decommissioning of a nuclear power plant (NPP) generates various types of waste and approximately 70% of the waste is reported to be concrete waste [1,2,3,4,5]. Concrete waste from NPPs is characterized by being heavy and volumetric, and thus requires a larger area for its disposal. However, in the Republic of Korea, the cost for the disposal of 200 L intermediate or low-level waste is around 13,000 USD [3,6] which is approximately three times more expensive than the disposal cost in the United States of America. For this reason, the reduction in the amount of concrete waste has become a “must do” for the Republic of Korea.

When concrete is exposed to high levels of radiation, many different elements can be activated [7,8]. Most of them lose their radioactivity during the initial cool-down period in the NPP decommissioning process. The remaining radioactive elements that are of major concern are ^60^Co and ^152^Eu or ^154^Eu [9,10,11,12], and these elements are reported to reside in the hydrated cement paste rather than the aggregate [11,12,13]. This means, if the cement paste portion of concrete can be successfully removed from the surface of the aggregate, most of the aggregate can be processed as “Exempt Waste” [14,15] to reduce the total amount of radioactive concrete waste generated from the decommissioning of NPP. The remaining powder that is separated from the aggregate will need to be solidified and stored in a designated disposal facility.

In our previous work [16], aggregate in concrete was heat treated for efficient separation of cement paste, and the separated cement paste portion of the waste (recycled cement powder) was utilized as a binder for the immobilization of the liquid radioactive waste simulant. Recently, achieving the requirements for the circular economy principles have been strongly emphasized to meet CO_2_ neutral era of cement-based materials [17]. Heat treatment is an accomplishable approach because hydrated cement paste and aggregate have different thermal expansion coefficients, and thus create more cracks in the interface for efficient separation [15,18,19]. There are a number of studies [18,19,20] that have mentioned hydrated cement paste with proper heat treatment had recovered hydraulic properties to produce good mechanical strength. Using such treatments, the results from our previous work [16] exceeded the criteria of 28 day compressive strength, thermal cycling, and leachability required for wasteform to be stored in a disposal facility. Recycling of activated cement powder is important because it will reduce the amount of binder required for immobilization of other radioactive waste (by full or partial replacement), which will eventually achieve the objective of “zero concrete waste generated from NPP”. 

It should also be noted that our previous work [16] used the equipment setup which applied the heat treatment process prior to the separation of recycled cement powder. The original purpose was to completely remove the cement paste from the aggregate. However, this original separation process required a large volume furnace for heat treatment since all the crushed concrete needed to be heat treated. The original process was found to be less efficient (especially in terms of energy), and thus a major change was made to locate heat treatment processes after the separation of recycled cement powder from aggregates. These changes reduced the amount of material to be heat treated, but also made recycled cement powder contain larger amounts of aggregate particles because a higher mechanical impact was necessary to be applied to increase the separation rate. Since the amount of inert aggregate particles reduces the reactivity of recycled cement powder, some additional experimental works were designed to verify whether this recycled cement powder could be applicable for the immobilization of liquid radioactive waste or not. 

This work was designed to evaluate the performance of recycled cement powder, obtained from a modified separation system, as a solidifying agent for the immobilization of radioactive waste. If radioactive recycled cement powder can be used to solidify other types of radioactive waste, the amount of radioactive waste generated from concrete will be conceptually “zero”, so this approach could create a significant environmental and economic impact on society. For experiments, concrete with more than 1 year of age was used for the separation process of fine and coarse aggregates, and recycled cement powder was used as a binder to make wasteform specimens. To understand the effect of temperature in heat treatments, recycled cement powder was heat treated at 600 and 700 °C and used to prepare various wasteform specimens to measure compressive strength and leachability of wasteforms. The 1 M CsCl solution was used as a liquid waste simulant. Since it was difficult to use radioactive materials due to safety issues, nonradioactive surrogates were used during entire study. 

## 2. Experimental Procedure 

### 2.1. Concrete Waste

In order to simulate the concrete waste generated during the decommissioning process of a nuclear power plant, a nonradioactive concrete specimen was prepared using the mix proportions shown in Table 1. The mix design of the source concrete followed one of the possible candidate concrete mix designs [21,22] used for the construction of Kori-1 unit. Ordinary type I Portland cement with a Blaine surface area of 3542 cm^2^/g and a density of 3.13 g/cm^3^ was used. Crushed stone with a bulk density of 2.6 g/cm^3^ and a nominal maximum size of 19 mm was used as a coarse aggregate. Standard sand from the International Organization for Standardization (ISO) with a bulk density of 2.6 g/cm^3^ and fineness modulus of 2.8 was used as fine aggregate. After mixing, the imitated concrete for Kori-1 unit was cured in ambient air for a year. 

### 2.2. Recycled Cement Powder

One-year old concrete specimens were jaw-crushed into 50–75 mm sized fractions and placed into a roll crusher. Roll crushing processes were operated at 300 rpm for 20 min. The size of the concrete was reduced to 25 mm or smaller and crushed aggregate was placed into the vibration mill. In the vibration mill, aggregates were vibrated for about 30 min at 3600 vpm to move through a narrow path whose height was close to 25 mm (slightly higher than 25 mm). Part of the concrete that was separated during this process was screened using 150 µm sieve. The powder that passed through 150 µm sieve was placed into a furnace for heat treatment.

During this process, most of the concrete which experienced the vibration process was moved to the separator. The separator had a 20° angle inclination so that the materials could easily move down when 980 rpm was applied. In the separator, concrete fractions moved down to the 1 mm sieve, and were separated using a 150 µm sieve. Materials that passed through the 150 µm sieve were moved into the furnace for heat treatment. The separation process was repeated until almost no recycled cement powder was obtained. 

It should be noted that previous works [16] have used a heat treatment temperature of 600 °C for 2 h because the temperature range of 600–700 °C was reported to maximize the reactivity of recycled cement powder [18,19,20,23,24]. However, there are some existing arguments on the optimum temperatures for recycled cement powder, which was considered to be 700 °C rather than 600 °C [25,26]. Therefore, in this work, the temperature for heat treatment was performed at 600 and at 700 °C. Heat treatment time was set to 1 and 2 h to check the possibility of reducing the heat treatment time from 2 h to 1 h.

### 2.3. Characterization of Recycled Cement Powder 

Chemical compositions of recycled cement powder after heat treatment at each condition were analyzed using a wave dispersive X-ray fluorescence spectrometer (XRF-1800, Shimadzu, Kyoto, Japan). An X-ray diffractometer (Ultima IV, Rigaku, Tokyo, Japan) was used to study mineralogical changes after heat treatment. The surface area of the recycled cement powder was also measured using N_2_ gas adsorption (BET) (TriStar II 3020, Micromeritics, Norcross, GA, USA). 

### 2.4. Preparation of Wasteform Specimen Using Liquid Waste Samples

Cylindrical wasteform specimens with dimensions of 1.5 cm (diameter) × 3.0 cm (height) were prepared by mixing recycled cement powder and waste simulant solutions. Nonradioactive 1 M CsCl (Cesium chloride, Sigma-Aldrich, Inc., St. Louis, MO, USA) solution was used as a waste simulant. Solution to binder ratio (S/B) of the wasteform was set to 0.5. Specimens made of deionized water were also prepared for comparison purposes. It should be noted that nonradioactive surrogates were used during entire experimental program. 

For mixing, each ingredient was added to a plastic bowl and hand mixed using a steel spatula. The dispersion of the wasteform was not as stiff as what was observed from previous work [16], so commercial superplasticizer was not used, to prevent the impact caused by superplasticizer on the leachability of the Cs. After the hand mixing was finished, the mixture was cast into the mold. The mold was rodded and vibrated to consolidate the wasteform mixture and covered by parafilm for one day at a 23 ± 2 °C to prevent evaporation of the moisture. After one day, the wasteform specimens were demolded and stored in sealed moist conditions for 28 days. During curing, the specimens were not in contact with water. The mix proportions of the wasteform specimens are summarized in Table 2.

### 2.5. Hydration Study

The hydration characteristics of the recycled cement powder that was mixed with a deionized water and 1 M CsCl solution was investigated using an 8-channel isothermal calorimeter (TAM AIR, TA Instruments, USA). Immediately after the mixing of the wasteform, approximately 4.5 g of the sample was placed into the glass vial, covered by rubber cap, and carefully inserted into the measurement channel in the calorimeter. The isothermal calorimetry measurements were carried out at 21 °C for a week. XRD analyses were also performed on the 28-day-old wasteform specimens to investigate the phase compositions after the reaction of recycled cement powder with the 1 M CsCl solution.

### 2.6. Compressive Strength

At 28 days, the compressive strengths of the wasteform specimens were measured using a compression testing machine (WJ-100S, Woojin Co. Ltd., Seoul, Korea). The test procedure basically followed the ASTM C 39 standard test method for compressive strength of cylindrical concrete specimens [27] with a slight modification in the loading rate. The loading rate of the measurement was set at 1 mm/min. The loading rate was set a bit slower than the loading rate designated in ASTM C 39 to reduce the effect caused by the smaller size of the specimen.

### 2.7. Thermal Cycling

A thermal cycling test was performed on wasteform samples with 28 days of age. An expired specification, the ASTM B 553 [28] standard test method for thermal cycling of electroplated plastics, has been widely used to evaluate wasteform integrity against thermal impacts. Thermal cycling test procedures consist of 30 cycles (highest temperature of 60 °C and lowest temperature of −40 °C) within a 10 day time period: from 20 °C to 60 °C (one hour), at 60 °C (one hour), from 60 °C to 20 °C (one hour), at 20 °C (one hour), from 20 °C to −40 °C (one hour), at −40 °C (one hour), from −40 °C to 20 °C (one hour), and at 20 °C (one hour) to complete total of 8 h cycle. After completing the thermal cycling test performed for 10 days, the wasteform specimens were subjected to compressive strength tests using the ASTM C 39. The same loading rate (1 mm/min) was used for compressive strength measurements after thermal cycling.

### 2.8. Leachability

Cs leachability from wasteforms was evaluated using the ANSI/ANS 16.1 measurement of the leachability of solidified low-level radioactive waste by a short-term test procedure [29]. Wasteform specimens of 1.5 cm (diameter) and 3 cm (height) were loosely held using plastic wire (to maximize the contact area with the deionized water) and submerged into deionized water at a liquid volume to solid surface area ratio of 10 mL to 1 cm^2^. Leachate solution was extracted using a 0.45 μm syringe filter at 2 and 7 h and at 1, 2, 3, 4, 5, 19, 47, and 90 day. After the sampling of leachate, the deionized water was replaced and lids were covered to prevent the evaporation of water from the container during the leaching process. Concentrations of Cs in leachate solution were analyzed using ICP MS (7900 ICP-MS, Agilent, Santa Clara, CA, USA). After the termination of the 90 day leaching test, the compressive strength of wasteform was also measured.

The diffusivity of Cs was calculated using the Equation (1) for simple radial diffusion from a cylinder to an infinite bath.

(1) $$D=\pi {\left[\frac{a_{n}/A_{o}}{{\left(\Delta t\right)}_{n}}\right]}^{2}{\left[\frac{V}{S}\right]}^{2}T$$

where *D* is the effective diffusivity (cm^2^/s); *a_n_* is the quantity of a nuclide released from the specimen during the leaching interval *n*; *A_o_* is the total quantity of a given contaminant in the specimen at the beginning of the first leaching interval (i.e., after the initial 30 s rinse); *V* is the volume of the specimen (cm^3^); *S* is the geometric surface area of the specimen as calculated from the measured dimension (cm^2^); *T* is the leaching time representing the “mean time” of the leaching interval (s).

From calculated diffusivity, the leachability index (LI) of Cs was calculated using Equation (2). According to Equation (2), the lower diffusivity indicates a higher LI.

(2) $$\mathrm{LI}=L_{i}=-\log\left(\frac{D}{{\mathrm{cm}}^{2}/s}\right)$$

## 3. Results

### 3.1. Characteristics of Recycled Cement Powder

#### 3.1.1. Chemical Composition

Chemical compositions of recycled cement powder which were heat treated at 600 and 700 °C for 1 and 2 h are presented in Table 3. As shown from Table 3, the amount of oxygen decreased as the heat treatment temperature increased. The reduction in oxygen content was associated with the partial decomposition of CaCO_3_ into CaO and CO_2_. AN increase in Ca content of recycled cement powder at 700 °C was also associated with partial decomposition of CaCO_3_.

#### 3.1.2. Mineralogy

**Figure 1:**
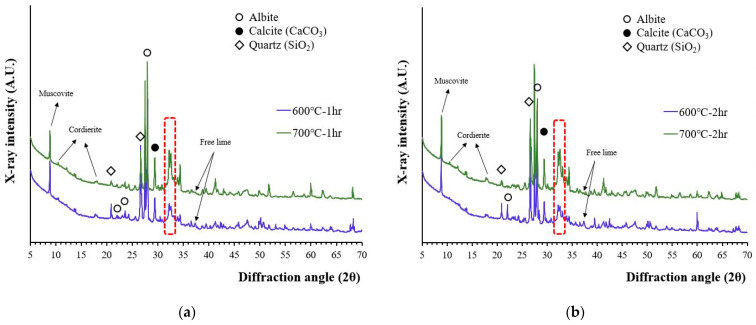
The XRD patterns of recycled cement powder heat treated at 600 and 700 °C: (**a**) for 1 h; and (**b**) for 2 h.

The X-ray diffraction patterns of recycled cement powder which were heat treated at 600 and 700 °C for 1 and 2 h are presented in Figure 1. According to Figure 1, the peaks that originated from the aggregate (quartz, albeit, muscovite, etc.) affected the characterization of cement related phases. The 2θ peak at 18.01°, which was suspected to be portlandite, was still observed, but the 2θ peak at 18.01° can be better correlated with cordierite because the main peak of portlandite (2θ at 34.10°) was not observed and 600 °C is the maximum temperature range that decomposition of Ca(OH)_2_ is completed. A low-intensity free lime (2θ of 37.35) peak was also observed meaning that partial decomposition of calcite occurred at this temperature.

It should be noted that the XRD peaks around a 31–34° 2θ diffraction angle can be corelated with a phase change caused by the dehydration of calcium silicates [16,30]. As temperature increased from 600 to 700 °C, the peak intensity at this region continued to increase. The crystallinity of this phase has increased as temperature for heat treatment increased although the peaks were still wider and lower compared to other crystalline phases originated from aggregates. The lime peak also exists at 700 °C, but not all calcite has been decomposed because the full decomposition of calcite is reported to occur above 800 °C [31].

#### 3.1.3. Surface Area

The surface area data of the recycled cement powder from the BET analyses are summarized in Table 4. The surface area of Portland cement was included for the purpose of comparison. All recycled cement powder showed higher surface areas than Portland cement and they were associated with the decomposition of chemically bound water from hydration products (mostly C-S-H). The space that was originally occupied by chemically bound water became empty spots and created additional surface areas in recycled cement powder.

As the temperature increased from 600 to 700 °C, a reduction of approximately 50% of surface area was observed. A drop in surface area can be associated with various explanations, but in this case, it is better to correlate it with the recrystallization of amorphous silicate. In general, crystallization of amorphous silicate was reported to occur at a temperature above 725 °C [32,33,34,35], but there were other reports that mentioned the transition starting to occur at a temperature range between 600 and 700 °C [36,37,38]. The results from this work suggests that the partial crystallization of dehydrated calcium silicates has occurred during the heat treatment of recycled cement powder at 700 °C, and this was evidenced by the reduction in the surface area (closing empty pore due to recrystallization) as well as the increase in XRD peak intensity at around 31–34°.

### 3.2. Reaction of Recycled Cement Paste

**Figure 2:**
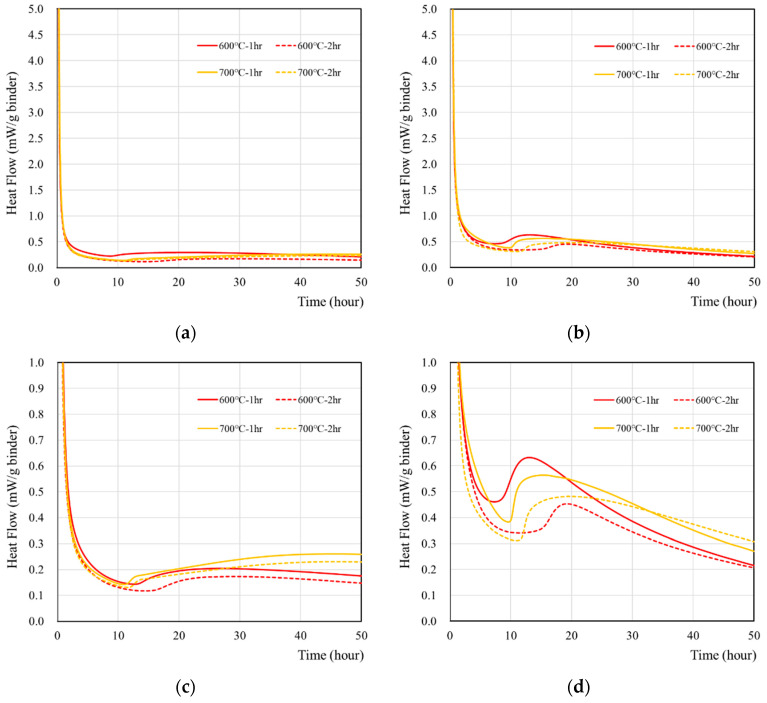
The heat flow of wasteform containing various types of liquids: (**a**) with deionized water, (**b**) with 1 M CsCl solution, (**c**) with deionized water, and (**d**) with a 1 M CsCl solution. Note that (**a**,**b**) were redrawn in the heat flow scale of 0–1.0 mW/g binder to facilitate the analyses.

The hydration characteristics of wasteforms made of deionized water and recycled cement powder that were heat treated at 600 and 700 °C for 1 and 2 h are presented in Figure 2. According to Figure 2a, the reaction of recycled cement powder with deionized water generated the majority of the heat flow at the time of mixing and became stabilized after around 1–2 h. When a 1 M CsCl solution was used (Figure 2b), the same initial high-intensity peak was observed, but the time to achieve stabilization took slightly longer (2–3 h). In addition, the peak at around the 10–40 h period was approximately two times higher than with deionized water (Figure 2c,d). In general, recycled cement powder with a 1 h heat treatment showed a slightly higher peak than 2 h heat treatment, but the effect was minimal.

**Figure 3:**
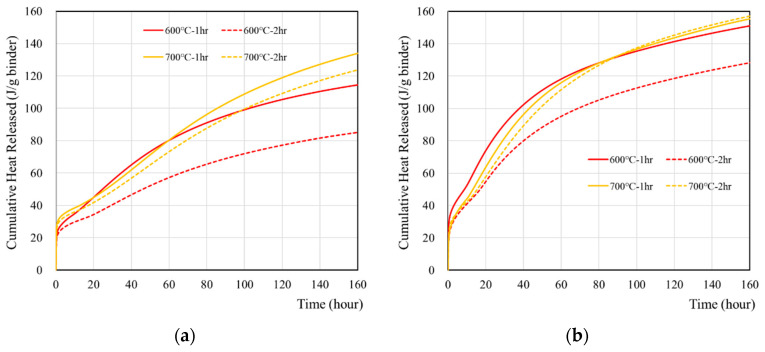
Cumulative heat of wasteforms containing various types of liquids: (**a**) with deionized water, (**b**) with 1 M CsCl solution.

The cumulative heat of wasteforms with various simulant solutions were measured for seven days and are presented in Figure 3 and Table 5. The sample with 1 M CsCl showed higher heat than the sample with deionized water. The highest heat was observed from 700 °C samples, regardless of using 1 M CsCl, and it was associated with the hydration of free lime. It is interesting to note that cumulative heat flow curve of recycled cement powder did not show any plateau for a seven-day time period. This is quite different from the behavior of ordinary Portland cement [39,40,41] which showed a plateau with the same amount of reaction time. It seems that the reaction of recycled cement powder may occur with the high-initial-heat evolution at the time of mixing with a slower but gradual heat evolution for a longer period. However, further investigation (e.g., the long-term monitoring of heat, hydration, and adiabatic temperature rise, and long-term strength) is necessary in order to verify this hypothesis.

**Figure 4:**
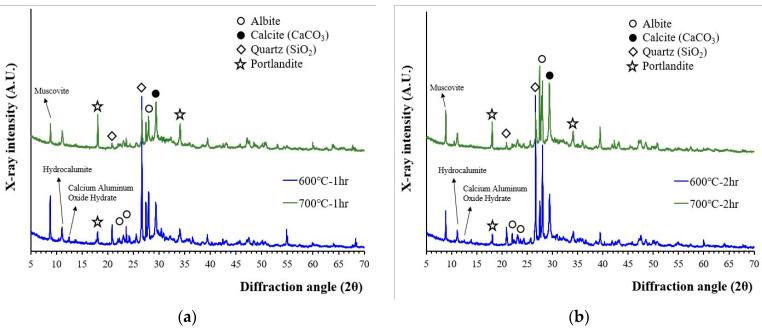
XRD patterns of wasteform specimens made of a 1 M CsCl solution and recycled cement powder that was heat treated at 600 and 700 °C: (**a**) for 1 h; and (**b**) for 2 h.

The XRD patterns of wasteform specimens after their reaction with the 1 M CsCl solution are presented in Figure 4. The XRD patterns of the wasteform specimens showed an increase in portlandite peak intensity but did not show any significant differences in terms of mineralogy whether recycled cement powder was heat treated at 600 or at 700 °C. A higher portlandite peak was associated with a reaction of free lime. As observed from Figure 1, the wasteform specimens after reaction with the 1 M CsCl solution still contained aggregate related phases such as quartz, albeit, and muscovite. However, from Figure 4, the wasteform specimen also contained hydration phases such as portlandite and hydrocalumite. It should be noted that the formation of hydrocalumite (chloride bearing calcium aluminum layered double hydroxide) was induced by the 1 M CsCl solution, meaning that chloride ion was captured within the layered structure of the calcium aluminate phase. Although it is uncertain where Cs was captured, it is highly likely to be located within the calcium silicate hydrate structure by taking spots of alkali and alkaline earth metals (such as Ca, Mg, Na, and K) that play the role of a charge balancing cation within silicate structure.

It is also interesting to notice that no CsCl peaks were observed with the 1 M CsCl solution. This finding is important because the recycled cement powder with 3 M CsCl (from our previous work [17]) showed the presence of crystalline CsCl. When a 3 M CsCl solution was used (in previous work), Cs and Cl exceeded their holding capacity of recycled cement powder, and uncaptured Cs and Cl were crystallized during the hardening process. With the 1 M CsCl solution, Cs and Cl did not exceed the maximum holding capacity of recycled cement powder, and therefore CsCl was not crystallized. The result indicates that Cs can be successfully captured within the structure of the hydration phases up to at least the 1 M CsCl concentration although the maximum holding capacity of Cs and Cl within the structure of recycled cement powder is still unknown.

### 3.3. Compressive Strengths

**Figure 5:**
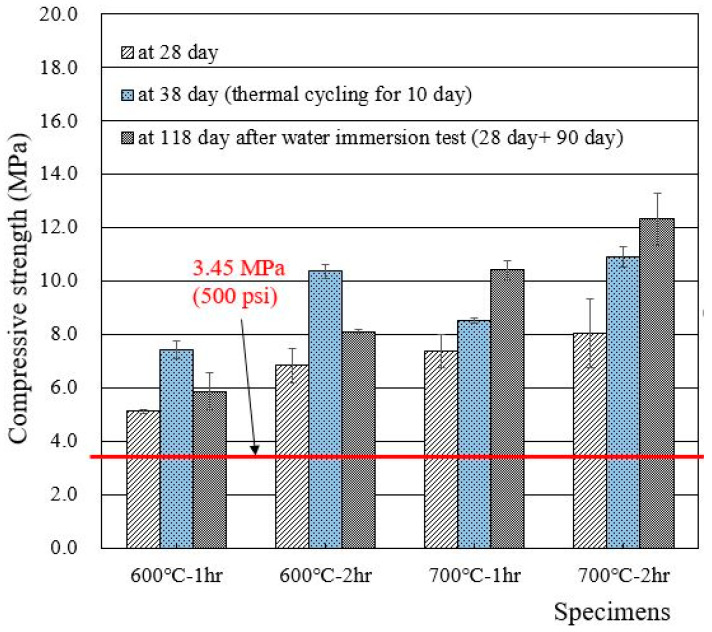
Compressive strengths of wasteform specimens made of deionized water and recycled cement powder that were heat treated at 600 and 700 °C.

Compressive strengths of cylindrical specimens made of deionized water and recycled cement powders that were heat treated at 600 and 700 °C for 1 and 2 h are presented in Figure 5. The 28 day compressive strength of wasteforms with deionized water met the acceptance criteria for waste disposal (3.45 MPa), showing 5–8 MPa. The increase in heat treatment temperature and time seemed to increase the compressive strength of the wasteform specimen. Interestingly, the strength after thermal cycling was found to be higher than the 28 day compressive strength. The reason for such an increase is unclear, but, at least, the specimen was not damaged during the 10 day thermal cycling test. In general, the specimen that was immersed in water for 90 additional days showed a higher strength than the 28 day strength due to the facilitation of hydration.

**Figure 6:**
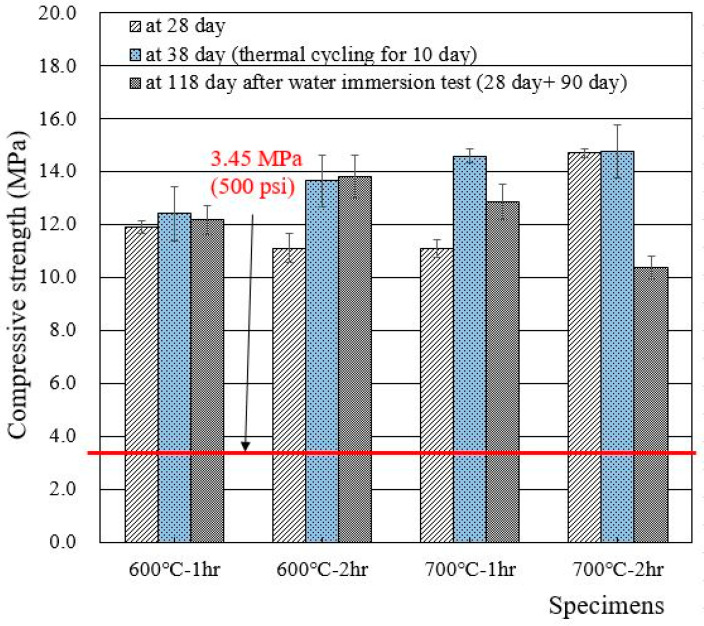
Compressive strengths of wasteform specimens made of 1 M CsCl and recycled cement powder heat treated at 600 and 700 °C.

When the 1 M CsCl solution was used to make wasteform specimens, the 28 day compressive strength increased from 5 to 8 MPa (with deionized water) to 11–15 MPa, as shown in Figure 6. It is clear that the use of 1 M CsCl increased the compressive strength of the wasteform, but it is still unable to designate which specific element (Cs or Cl) was the reason for the increase in compressive strength. Additional research is required to clarify the reason. Similar to the case of Figure 5, thermal cycling did not cause any damage to the wasteform specimen with 1 M CsCl.

### 3.4. Leachability

Leachability indices of Cs from wasteforms using the ANS/ANSI 16.1 leaching test procedure are summarized in Table 6. The leachability indices of Cs exceeded the LI value of six and met the acceptance criteria required for wasteform disposal. The leachability indices of Cs ranged from 8.63 to 13.39 during measurement. The leachability indices of Cs exceeded the LI value for acceptance criteria (LI of six). The initial low LI value indicated a higher amount of leaching at an early leaching time which was associated with the leaching of the surface located elements [42]. With a longer leaching period, the LI values started to increase and became stabilized as leaching intervals increased. In general, the wasteform showed a proper leaching performance against Cs.

It should be noted that the initial LI values obtained from the wasteform ranged from 8.63 to 9.07. These values were higher than the LI values of 6.65–7.06 that were observed from our previous work [16]. Lower LI values obtained from our previous work were associated with the presence of CsCl crystal that was not captured within the structure of recycled cement powder after hydration. As mentioned from the XRD analyses, this was related to the excessive concentration of the CsCl solution (3 M) [16] that was used to make wasteform specimens. When CsCl crystal was in contact with water, it readily dissolved and increased the concentration of Cs in the leachate, thereby reducing the LI value of Cs.

It is also important to note that the final LI values obtained from the wasteform ranged from 10.71 to 13.39. These values were lower than the final LI values observed from our previous work which ranged from 15.29 to 15.30 [16] and were associated with the higher surface area of recycled cement powder used in the previous work. When the same heat treatment process (600 °C for 2 h) was applied for recycled cement powder, the specific surface area (by BET) decreased from 12.37 m^2^/g (previous work) [16] to 7.07 m^2^/g (as reported in Table 4). When the surface area was higher, the dissolved Cs could be more effectively adsorbed within the empty spaces of amorphous silicate structure that were created by the decomposition of water during heat treatment. Additional hydration followed where cations were located and the voids were closed. Such mechanisms eventually brought a complete sequestration of Cs that needed to be immobilized in the wasteform.

## 4. Discussion

As reported earlier, the recycled cement powder used in this work had a major difference compared to that used in our previous work [16]. In previous work, heat treatment was applied prior to the separation process to maximize the separation efficiency, so it was evaluated as a costly approach. However, in this work, heat treatment was applied after the separation process in order to increase energy efficiency as it was found that such a change has affected the properties of recycled cement powder quite significantly. Comparing the properties of recycled cement powder that was heat treated at 600 °C for 2 h, the specific surface area decreased from 12.37 m^2^/g to 7.07 m^2^/g. This was mainly associated with the increase in the amount of crystalline aggregate powder that was included during separation process. The reduction in surface area significantly affected the solution to binder ratio of the wasteforms because recycled cement powder with higher surface areas requires higher amount of water for suitable workability.

For this reason, a high solution to binder ratio (S/B of 0.7) had to be used in previous works [16]. Some amount of polycarboxylate based superplasticizer also had to be used to provide sufficient flowability for casting the wasteform specimens. In this work, a relatively low solution to binder ratio (S/B of 0.5) could be used. No superplasticizer was required because the fresh mixture was quite workable for casting. The reduction in solution to binder ratio (identical to the water to cement ratio) increased the compressive strength of the wasteform from 5.2 MPa (with 3 M CsCl solution in previous work) to 11.9 MPa (with 1 M CsCl solution in this work). However, it should be noted that a higher aggregate portion in recycled cement powder will reduce the amount of reacting material, so an increase in the amount of aggregate particle in recycled cement powder should always be monitored accurately.

Although the increase in surface area of recycled cement powder played a negative role in workability, it also played a positive role in leachability. With a reduction in surface area (in this work), the final LI values of Cs from the wasteform (LI of 13.20) was lower than what was observed from previous work (LI of 15.30). This indicates that controlling the surface area of recycled cement powder is a key parameter. According to Table 7, a summary that has been found regarding the differences between the recycled cement powder used in this work and the previous work is that heat treatment after separation has advantages in energy efficiency and workability, whereas separation after heat treatment (used in the previous work) has advantages in leachability and waste loading (by using higher amounts of liquid simulant). However, it should be noted that the increase in compressive strength that was observed in this work was associated with the reduction in S/B, not with the increase in aggregate portion of recycled cement powder because the increase in aggregate proportion would eventually decrease the compressive strength of the wasteform. The efforts to find the maximum capacity of aggregate particles that can be included in recycled cement powder, without the help of Portland cement blending for solidifying other types of radioactive waste, are under progress in order to suggest the optimum operation condition of the separation processes used in our previous and current works.

The effect caused by 600 °C and 700 °C heat treatment showed some differences. The surface area after the heat treatment was higher for 600 °C, but the heat evolution after hydration was higher for 700 °C. Higher heat evolution was associated with a rapid hydration of free lime to form portlandite. However, it should be noted that this reaction also brought stiffer flowability during mixing although the surface area was higher for 600 °C than for 700 °C. In terms of compressive strength, the specimen with deionized water showed a higher strength with 700 °C, but the difference became minimal when recycled cement powder was reacted with a 1 M CsCl solution. No clear difference in Cs leachability was observed. Considering all observed results and energy efficiency, the authors believe that heat treatment at 600 °C is a better option than heat treatment at 700 °C.

The reason why incorporation of 1 M CsCl increased compressive strength of wasteforms was not clearly identified. Since it is generally known that the addition of chloride in the Portland cement-based system accelerates early hydration with reduction in long-term strength [43,44], it is more reasonable to suspect Cs as the main reason for the increase in compressive strength. The authors have observed the same effect from all experimental cases with recycled cement powder (also from the results that have not yet been published), but it is still uncertain whether such effect also occurs with ordinary Portland cement system or not. More systematical experimental works are in progress in order to reveal strength enhancement caused by Cs.

## 5. Conclusions

In this work, recycled cement powders that experienced 600 °C and 700 °C heat treatment for 1 h and 2 h were used to immobilize Cs containing liquid waste simulant. The performance of recycled cement powder as a solidifying agent was evaluated using a 28 day compressive strength, thermal cycling, and 90 days leaching text. The following conclusions are derived from the experimental results.

1. The recycled cement powder used in this work (experienced heat treatment after separation process) met the wasteform acceptance criteria. The effects caused by heat treatments were also observed, and heat treatment at 600 °C was found to be a better option than heat treatment at 700 °C.
2. Recycled cement powder used in this work required less water demand for mixing compared to the recycled cement powder used in the previous work (experienced heat treatment prior to the separation process). This was mainly related to the reduction in the surface area due to a higher inclusion of crystalline aggregate powder.
3. A reduction in surface area enabled a lower solution to binder ratio (S/B 0.5) of wasteform, and, as a result, a relatively higher compressive strength was obtained compared to the case observed from previous work which used a higher solution to binder ratio (0.7).
4. A reduction in surface area seemed to be a reason for lower final leachability indices of Cs from the wasteform.
5. Application of heat treatment after complete separation of recycled cement powder can be an applicable alternative for the immobilization of liquid waste. However, when using such an approach, the amount of aggregate particle in recycled cement powder should always be accurately monitored because it will eventually reduce the compressive strength of the wasteform.
6. Separation of recycled cement powder from concrete and its utilization as a solidifying agent for immobilization of other radioactive waste is a way to achieve “zero” waste production (in an ideal situation) during the decommissioning of concrete in a nuclear power plant.

## Figures and Tables

**Table 1:** Mixing details of nuclear power plant concrete.

Type	Strength (psi)	W/B	S/a (%)	Unit Contents (kg/m^3^)				Admixture (mL)	
				Water	Cement	Fine Aggregate	Coarse Aggregate	WRA	AEA
Concrete	3000	0.621	45	164.93	265.79	806.26	983.06	482.33	18.39

**Table 2:** Test protocol for wasteform specimens with recycled cement powder.

Recycled Cement Powder (Heat Treatment Time)		S/B	Mixing Water (Simulant Solution)
Temperature	Time		
600 °C	1 h, 2 h	0.5	DI-water CsCl 1 M
700 °C	1 h, 2 h		

S/B indicates solution (waste simulant solution) to binder (recycled cement powder) ratio.

**Table 3:** Chemical compositions of recycled cement powder after heat treatment at 600 and 700 °C for 1 and 2 h.

	Ca	Si	Al	Fe	K	Mg	S	Na	Ti	P	Mn	Zn	Sr	O
600 °C 1 h	40.47	14.01	2.63	1.03	1.38	1.70	0.61	0.69	0.21	0.05	0.04	0.04	0.03	37.07
600 °C 2 h	38.58	15.26	2.70	0.97	1.44	1.68	0.58	0.68	0.21	0.04	0.04	0.04	0.03	37.72
700 °C 1 h	44.44	11.69	2.43	0.98	1.15	1.82	0.72	0.51	0.19	0.06	0.04	0.04	0.03	35.88
700 °C 2 h	43.99	12.01	2.42	0.98	1.17	1.81	0.73	0.43	0.21	0.05	0.04	0.04	0.02	36.07

**Table 4:** Summary of results from N_2_ gas adsorption (BET).

Specimens		Surface Area			Pore Size
		SA_BET_ (m^2^/g)	SA_Micro_^(a)^ (m^2^/g)	SA_Ext_ (m^2^/g)	PS_Dia_^(b)^ (Å)
**Portland Cement**		1.2189	0.2506	0.9683	196.2565
Recycled cement powder	600 °C-1 h	7.4878	1.5073	5.9805	28.9918
	600 °C-2 h	7.0737	1.5904	5.4832	35.5834
	700 °C-1 h	3.7347	1.0372	2.6975	31.7818
	700 °C-2 h	4.0215	0.9757	3.0458	36.1131

Analysis adsorptiove: N_2_ (77.300 K); molecular cross-sectional area: 0.1620 nm^2^. ^(a)^ SAMicro (m^2^/g): SABET-SAExt. ^(b)^ PSDia = pore size (Å): The pore diameter showing is the max.

**Table 5:** The seven-day cumulative heat flow of wasteform made of recycled cement powder and 1 M CsCl solution.

No.	Specimen	Cumulative Heat Flow (J/g Cement)	
		Di-Water	CsCl 1 M
1	OPC	359.62	323.34
2	600 °C-1 h	115.83	145.24
	600 °C-2 h	86.30	125.41
3	700 °C-1 h	136.077	157.18
	700 °C-2 h	125.83	155.26

**Table 6:** Leachability Index of Cs from cylindrical specimens.

Leaching Time (Days)	LI			
	600 °C-1 h	600 °C-2 h	700 °C-1 h	700 °C-2 h
0.083	8.63	8.80	8.90	8.87
0.292	8.73	8.88	9.08	9.07
1	8.80	8.90	9.17	9.17
2	9.06	9.09	9.28	9.25
3	9.01	9.28	9.42	9.41
4	9.47	9.44	9.46	9.49
5	9.69	9.60	9.52	9.56
19	10.75	10.44	10.01	10.04
47	10.54	10.82	10.43	10.84
90	13.39	13.20	10.71	11.55

**Table 7:** A comparison of the properties of recycled cement powder used in previous work and this work.

	In Previous Work		In This Work
Heat Treatment	Prior to the Separation Process		After Separation Process
Separation process	1. Jaw crushing to separate coarse and fine aggregates 2. Heat treatment of aggregate (600 °C 2 h) 3. Milling without ball (240 rpm, 22 h) 4. Recovery of cement powder less than 150 μm		1. Jaw crushing and roll crushing 2. Powder separated using 150 μm sieve after vibration 3. Heat treatment (600 °C 2 h)
The specific surface area	12.37 m^2^/g		7.07 m^2^/g
Solution to binder ratio (S/B)	0.7		0.5
Solution type	CsCl 3 M solution		CsCl 1 M solution
Superplasticizer	none	0.67 % by wt. of binder	none
Compressive strength at 28 days	5.20 MPa	6.36 MPa	11.9 MPa
Final LI values	15.30	15.29	13.20
